# Genetic Susceptibility of the Host in Virus-Induced Diabetes

**DOI:** 10.3390/microorganisms8081133

**Published:** 2020-07-27

**Authors:** Keiichiro Mine, Yasunobu Yoshikai, Hirokazu Takahashi, Hitoe Mori, Keizo Anzai, Seiho Nagafuchi

**Affiliations:** 1 Division of Metabolism and Endocrinology, Faculty of Medicine, Saga University, 5-1-1, Nabeshima, Saga 849-8501, Japan; takahas2@cc.saga-u.ac.jp (H.T.); sunrisebyebyemoon@yahoo.co.jp (H.M.); akeizo@cc.saga-u.ac.jp (K.A.); 2 Division of Host Defense, Medical Institute of Bioregulation, Kyushu University, 3-1-1, Maidashi, Higashi-ku, Fukuoka 812-8582, Japan; yoshikai@bioreg.kyushu-u.ac.jp; 3 Liver Center, Saga University Hospital, Saga University, 5-1-1, Nabeshima, Saga 849-8501, Japan

**Keywords:** diabetes, virus, susceptibility genes

## Abstract

Enteroviruses, especially Coxsackie B viruses, are among the candidate environmental factors causative of type 1 diabetes. Host genetic factors have an impact on the development of virus-induced diabetes (VID). Host background, in terms of whether the host is prone to autoimmunity, should also be considered when analyzing the role of target genes in VID. In this review, we describe the genetic susceptibility of the host based on studies in humans and VID animal models. Understanding the host genetic factors should contribute not only to revealing the mechanisms of VID development, but also in taking measures to prevent VID.

## 1. Introduction

Diabetes mellitus is one of the most common metabolic diseases and the number suffering from it is increasing worldwide. The International Diabetes Federation (IDF) estimated that the prevalence of diabetes in those aged 20–79 years was 463 million globally in 2019. The number of patients was also forecasted to increase to 700 million by 2045 [1]. Diabetes results from reduced insulin secretion from pancreatic β-cells, insulin resistance in peripheral tissue, or both. These factors lead to hyperglycemia, which is associated with the development of chronic complications in many organs, including the eyes, heart, kidneys, nerves, and blood vessels. In 2019, the number of deaths due to diabetes was estimated to be 4.2 million globally [1]. Therefore, to eradicate diabetes, we have to take preventive and curative measures, and also elucidate the mechanisms behind its development.

Diabetes is classified by its etiology into four types: type 1, type 2, gestational diabetes, and specific types [2]. Type 1 diabetes (T1D) is caused by loss of insulin-producing pancreatic β-cells, leading to absolute insulin deficiency. Type 2 diabetes (T2D) is caused by progressive loss of insulin secretion from β-cells and/or insulin resistance. Gestational diabetes mellitus (GDM) involves cases in which there is no clear overt diabetes prior to gestation, but it then develops in the mother and is usually diagnosed in the second or third trimester of pregnancy. Specific types of diabetes are a result of other specific causes as follows: genetic defects, disease of the exocrine pancreas, endocrinopathies, drug- or chemical-induced diabetes, infections, uncommon forms of immune-mediated diabetes, and genetic syndromes sometimes associated with diabetes [3].

In both T1D and T2D, host genetic factors and environmental factors have been considered to be associated with the progressive loss of β-cell mass and/or functions [3,4]. Especially in the development of T1D, viruses have been suggested as one potential causal factor [5]. The first report suggesting the possible relationship between virus infection and T1D was published in *Boston Medical and Surgical Journal* by Harris in 1899 [6]. Subsequently, a number of viruses were reported to be associated with diabetes (Table 1).

Furthermore, in fulminant T1D, a clinical phenotype of T1D with abrupt onset and almost complete destruction of pancreatic β-cells, a viral contribution to the etiology is strongly suggested [7,15,32,33,34]. Viruses may contribute to the development of diabetes in several ways: direct β-cell destruction, triggering β-cell-specific autoimmunity, bystander damage via inflammation, bystander activation of T cells, molecular mimicry, and the induction of dedifferentiation [5,35,36]. It is clear that the pathogenesis of VID is complicated and can be considered to involve interplay between viral virulence and the host [5]. One reason for this is that even highly diabetogenic encephalomyocarditis virus (EMCV) induces diabetes only in selected strains of mice [21]. Although a number of studies on the mechanisms of the pathogenesis of VID have been performed, the exact mechanisms are still unknown. In this review, we focus on the role of host genetic factors in VID development, and present historic and new findings in the field of VID research.

## 2. Host Genetic Factors in Humans

In this section, host genetic factors in human VID are described. All of the Single Nucleotide Polymorphism (SNP) information was obtained from the Single Nucleotide Polymorphism Database (dbSNP). A summary of the observations described below is presented in Table 2.

### 2.1. IFIH1

Interferon induced with helicase C domain 1 (IFIH1), also known as Melanoma differentiation-associated gene 5 (MDA5), is a protein-coding gene that encodes the double-stranded RNA (dsRNA) recognition receptor (pattern-recognition receptor) and plays a role in antiviral immunity [45,46]. IFIH1 consists of the N-terminal tandem caspase-recruitment domains (CARDs), DExD/H-box helicase domains, and C-terminal domain (CTD). IFIH1 interacts with Mitochondrial antiviral signaling protein (MAVS) through their CARDs and starts the signaling cascade for the production of interferon [47]. A number of studies have reported that IFIH1 is associated with T1D [40,44,48].

#### 2.1.1. rs1990760

The single-nucleotide polymorphism rs1990760 (amino acid change, A946T; nucleotide change, C>T) is located in the CTD of IFIH1. It was reported that the variant is a gain-of-function mutation [49]. It was also reported that the variant does not affect IFIH1 activity [50]. The tyrosine allele is associated with increased risk for T1D [51] and other autoimmune diseases.

rs1990760 is linked with the high expression of type III IFNs but not with IFN-β in pancreatic islets from human donors following CVB3 infection ex vivo [39]. Studies have shown a trend for high viral titer and high expression levels of CVB3 RNA in islets carrying the risk allele. It was suggested that rs1990760 does not exert dramatic effects, but might have subtle effects on the ability to control viral replication [39]. In contrast to this finding, human peripheral blood mononuclear cells (PBMCs) carrying the rs1990760 SNP showed high basal and poly(I:C)-triggered production of IFN-β [51]. These observations imply that the effect of the SNP depends on the cell type. Mice with a knock-in mutation encoding rs1990760 exhibited improved survival to lethal viral infection, and the increased susceptibility to low-dose poly(I:C) induced autoimmune diabetes [51].

An analysis of subjects from a long-term clinical follow-up study in Finland (Finnish DIPP study) showed that the rs1990760 SNP is associated with the production of β-cell autoantibodies (IAA, GADA, IA-2A), but not the development of T1D [37]. In contrast, in Norwegian newborns, rs1990760 was reported not to be associated with the frequency of enterovirus RNA in stool samples and islet autoantibodies [38].

#### 2.1.2. rs35667974, rs35337543, rs35744605, and rs35732034

rs35667974 (I923V, T>C) is a missense variant located at exon 14 of IFIH1. rs35744605 (E627X, C>A,G,T) is a stop-gain or missense variant located at exon 10. rs35337543 (C>G,T) and rs35732034 (C>T) are splice variants located at introns next to exon 8 and exon 14, respectively. These four rare alleles are reported to be variants protective against the onset of T1D [51,52]. rs35667974 was also suggested as a T1D-associated locus in another study. Although these variants showed protective effects against T1D, rs35337543, rs35744605, and rs35732034 are loss-of-function variants, and the patients carrying these variants manifest extreme susceptibility to infections with common respiratory RNA viruses such as human respiratory syncytial virus (RSV) and human rhinovirus (HSV) in Australian and Switzerland children. [53]. In contrast, rs35744605, rs35667974, and rs35337543 were reported not to be associated with the frequency of enterovirus RNA in stool samples from Norwegian newborns [38]. In that study, there was a marginal increase in the prevalence of enterovirus RNA in those carrying rs35732034 [38].

#### 2.1.3. rs3747517, rs2111485, and rs13422767

rs3747517 (H843A, T>C) is a missense variant located at exon 13 of IFIH1. rs2111485 (A>G) and rs13422767 (G>A) are intergenic polymorphisms between IFIH1 and the fibroblast activation protein alpha (FAP) gene. These SNPs were reported to show an association with T1D in the Polish population [54]. Another study also indicated the association between rs2111485 and T1D [40]. Moreover, a family-based single-marker analysis indicated that the presence of cytosine at rs3747517 was associated with an increased risk of T1D [51].

### 2.2. TYK2

Tyrosine kinase 2 (TYK2), also known as JTK1, is a protein-coding gene that encodes the kinase associated with the cytoplasmic domain of type 1 and type 2 cytokine receptors. TYK2 belongs to the Janus kinase (JAK) protein family, and plays a role in the signaling of cytokines and growth factors, including type I and III IFNs, interleukin (IL)-6, IL-10, IL-12, IL-23, and fibroblast growth factor (FGF) 2 [55,56]. TYK2 consists of the FERM domain, SH2 domain, pseudokinase domain, and the C-terminal kinase domain [56,57]. Once activation of the cytokine receptors occurs, TYK2 is activated by auto- or trans-phosphorylation and activates the family of signal transducer and activators (STATs). TYK2 is reported to be associated with T1D and T2D, and also other autoimmune diseases such as systemic lupus erythematosus, multiple sclerosis, and hyper-IgE syndrome. The inhibition of TYK2 expression reduced the production of IFN-α and CXCL10, and induced apoptosis in human β-cells exposed to poly(I:C) ex vivo [58].

#### 2.2.1. rs2304256

rs2304256 (V362F, C>A) is a missense variant located in exon 8 that is part of the FERM domain. It was suggested that this SNP reduces interaction between TYK2 and interferon α/β receptor 1 (IFNAR1) [59,60]. The TYK2 gene expression levels increased modestly in whole blood and adrenal gland of those carrying the A allele [60]. There is evidence that the A allele of rs2304256 is a protective variant against T1D in those of European ancestry [61]. B lymphoblastoid cell lines (BLCLs) carrying the A allele showed lower IFN-α-induced Stat1 phosphorylation than those carrying the C allele [58]. A recent large-scale study using stool samples from children showed that rs2304256 is associated with the presence of the Enterovirus B group (EVB) in stools, but is not associated with the presence of islet autoantibodies (GADA, IA-2A, or IAA) in the United States and European populations [41]. Our previous study showed that there was no difference in the frequency of the rs2304256 SNP between T1D patients and healthy controls, and that rs2304256 is not associated with the presence of flu-like syndrome at T1D onset in the Japanese population [42].

#### 2.2.2. rs12720356

rs12720356 (I684S, A>C) is a missense variant located at exon 15, which is part of the pseudokinase domain. It was reported that rs12720356 did not alter TYK2 function [62]. In contrast, another study showed that this SNP led to reduced IL-12, IL-23, and type 1 IFN signaling-stimulated pSTAT4 response in CD4 and CD8 T cells [63]. A study using an immunochip also reported the association between rs12720356 and T1D [40]. Although rs12720356 appears to be associated with cytokine signaling, the association between this SNP and virus infection is unknown.

#### 2.2.3. rs2304258, rs17000728, rs17000730, rs2304259, rs891696485, and rs953883300

rs2304258 (C>T), rs17000728 (C>T), and rs17000730 (T>C) are 5′ UTR variants of the TYK2 gene. rs2304259 (T>G), rs891696485 (A>C/A>T), and rs953883300 (C>T) are intergenic polymorphisms between TYK2 and the cell division cycle 37 (CDC37) gene. These SNPs are in complete linkage disequilibrium in the Japanese and are thus called the TYK2 promoter variant (ClinVar, 440728) [42]. The TYK2 promoter variant showed reduced promoter activity of the TYK2 gene and an association with the risk of T1D; in particular, in patients with T1D it was associated with flu-like syndrome at diabetes onset and GADA-negativity [42]. It was suggested that T1D cases suggestive of a viral contribution to the onset show GADA-negativity, low IgE levels, and TYK2 promoter variant-positivity [43]. These findings imply that individuals carrying TYK2 variants may be a target of slow-acting pancreatropic viruses due to their dampened IFN response [64]. Surprisingly, TYK2 promoter variant also confers an increased risk of T2D with impaired insulin secretion activity, suggestive of partial β-cell damage due to potential viral infection [42,65].

### 2.3. CXADR

Coxsackievirus and adenovirus receptor (CXADR), also known as HCAR, is a protein-coding gene that encodes a transmembrane receptor containing two extracellular immunoglobulin-like domains, and thereby belonging to the immunoglobulin superfamily [66]. CXADR is a component of the tight junctions between the surfaces of cells [67]. CVB and subgroup C of the adenoviruses (Ad) use CXADR for entry into host cells [66]. It was reported that CXADR expression is enhanced in pancreatic islets from patients with T1D [68]. CXADR is highly expressed in pancreas tissue [66,69]. CVB infection was shown to result in decreased CXADR expression in β-cells [68,70].

#### 2.3.1. rs6517774

rs6517774 (A>G) is an intergenic polymorphism between CXADR and the long intergenic non-protein-coding RNA 1549 (LINC01549) gene. The G allele of rs6517774 was shown to be associated with a lower rate of detection of EVB in stools from United States and European children [41]. An association between this SNP and islet autoimmunity was also observed (the presence of GADA, IA-2A, or IAA) [41].

#### 2.3.2. rs2824404

rs2824404 (T>C) is a noncoding downstream transcript variant of CXADR. It was reported to be associated with the presence of islet autoimmunity in Finnish children [41].

### 2.4. BACH2

BTB domain and CNC homolog 2 (BACH2) is a protein-coding gene that encodes a transcription factor that belongs to the Cap‘n’Collar type of the basic region-leucine zipper factor family (CNC-bZip) [71]. BACH2 consists of a BTB/POZ domain, five cysteine–proline (CP) motifs, Zip, and a cytoplasmic localization signal (CLS). It is associated with the development of B cells, and the immune functions of DCs, B cells, and T cells [71]. Notably, BACH2 is a key regulator of CD4-positive T-cell differentiation that prevents abnormal inflammation by controlling the balance between the formation of Treg cells and effector CD4-positive T cells [72]. BACH2 is reported to be associated with T1D and also other autoimmune diseases [40,44,71,73]. The inhibition of BACH2 expression was shown to exacerbate cytokine-induced apoptosis in human β-cells ex vivo [74]. It was also reported that BACH2 restrains terminal differentiation to enable the generation of long-lived memory CD8-positive T cells and protective immunity against viral infection [75].

#### rs72928038, rs3757247, and rs11755527

rs72928038 (G>A), rs3757247 (C>T), and rs11755527 (C>G) are all variants located at introns of the BACH2 gene. The associations between these SNPs and T1D were reported from genome wide association study (GWAS) and immunochip studies [37,40,73]. rs3757247 demonstrated an association with an increased risk of GADA-positivity, while its association with the development of T1D was not observed in Finnish population (Finnish DIPP study) [37]. Although BACH2 is involved in antiviral immunity, its role in VID has not been investigated.

### 2.5. PTPN22

Protein tyrosine phosphatase non-receptor type 22 (PTPN22) is a protein-coding gene that encodes a class I protein tyrosine phosphatase (PTP). PTPN22 consists of three major domains: PTP domain, interdomain, and C-terminal domain [76]. PTPN22 is a negative regulator of signaling through the T-cell receptor (TCR) in T cells. Ptpn22-deficient mice were found to exhibit enhanced proliferation of T cells and T-cell-dependent Ig responses to antigen [77]. It was also reported that PTPN22 is associated with the regulation of Tregs, B cells, dendritic cells (DCs), and macrophages [76,78,79,80]. Therefore, PTPN22 plays an important role in immunoregulation and immune responses against infectious agents including viruses [76]. An association between PTPN22 and an increased risk of T1D has also been identified by GWAS [44,73,81]. For more information about PTPN22, see a previous review [76].

#### rs2476601

rs2476601 (R620W, C>T) is a missense variant located in exon 14, which is part of the C-terminal domain of PTPN22. CD4-positive T cells carrying rs2476601 from Hungarian children with T1D showed reduced activation, proliferation, IL-2 production, and intracellular calcium flux after anti-CD3/anti-CD28 or PHA stimulation ex vivo [80]. Although B cells carrying rs2476601 showed specific expansion of the transitional and anergic B-cell subsets, parallel changes in both BCR signaling and the composition of the B-cell compartment were observed in T1D patients and healthy controls irrespective of the PTPN22 genotype [79]. The rs2476601 SNP reduces toll-like receptor (TLR) signaling, TLR-driven type 1 IFN upregulation, and type 1 IFN-dependent activation in myeloid cells [78]; therefore, it was suggested that the possession of rs2476601 could contribute to the expression of autoimmunity, namely, defective myeloid cell capacity for type 1 IFN-driven suppression of inflammation in inflamed tissues [76]. A study that analyzed stools from the United States and European children who developed islet autoimmunity showed that prolonged shedding or consecutive positivity for EVB, human mastadenovirus C (HAdV-C), or HAdV-F was associated with rs2476601 [41]. Taking these findings together, T1D associated with rs2476601 may reflect virus infections that either directly destroy islets or prime maladaptive lymphocyte responses [76].

## 3. Host Genetic Factors in Animal Models

In this section, host genetic factors mainly in murine VID models are described. There are five available animal models of this type: EMCV-induced diabetes model in mice, Kilham rat virus (KRV)-induced autoimmune diabetes model in rats, CVB-induced acute autoimmune diabetes model in non-obese diabetic (NOD) mice, transgenic animal models in mice, and combined streptozotocin (STZ) and virus infection model in mice. Because the interplay between viruses and the host background is critical for the development of VID [5], the characteristics of these models should be described.

### 3.1. EMCV-Induced Diabetes Model

The highly β-cell-tropic and diabetogenic D variant of EMCV (EMCV-D) was isolated from the M variant of EMCV (EMCV-M) [21]. EMCV is a nonenveloped RNA virus that belongs to the Picornaviridae family, genus *Cardiovirus* [82]. This virus can replicate rapidly in vitro with a replication time of approximately 8 h [83]. Some strains of inbred mice, such as SJL, SWR, and DBA/2, develop diabetes within 5 days after EMCV-D infection through the intraperitoneal route [21,84]. In this model, the viral dose affects the mechanisms of diabetes development. Animals infected with a high dose of EMCV-D (1 × 10^5^ plaque-forming units (PFU)/mouse) develop diabetes mainly due to the replication of EMCV-D within β-cells and the destruction of the β-cells by viruses [85,86]. In contrast, animals infected with a low dose of EMCV-D (<1 × 10^2^ PFU/mouse) develop diabetes mainly due to inflammation caused by macrophages infiltrating into the islets [86,87,88].

In the high dose of EMCV-D infection model, T-cell- or B-cell-deficient mice failed to alter the incidence of diabetes onset; therefore, T-cell- and B-cell-mediated immune responses to the model appear to be negligible [85,86]. Anti-lymphocyte serum treatment also failed to prevent diabetes [85]. Even in the model infected with a high dose, macrophages could be detected in islets at 12–36 h after EMCV-D infection [87]. Anti-Mac-2 antibody treatment reduced the incidence of diabetes development in SJL mice [87]. Therefore, it was suggested that the replication of EMCV-D within the β-cells and the macrophages infiltrating into the islets could act synergistically to destroy β-cells, leading to the development of VID [86].

In the low dose of EMCV-D infection model, SJL mice—into which activated macrophages were transferred by silica treatment—showed an increase in the incidence of VID [89]. The depression of macrophages by their antibodies in turn resulted in a reduced incidence of VID [89]. The soluble mediators produced by macrophages such as interleukin (IL)-1β, tumor necrosis factor (TNF)-α, and nitric oxide (NO) play a central role in destroying β-cells in this model [88,90].

An intermediate dose such as 1 × 10^3^ PFU/mouse infection has also been used for analyzing the mechanisms of VID development. In this intermediate-dose model, EMCV-D appears to be sufficiently cytotoxic to destroy β-cells, as in the high-dose infection model [87]. T-cell- or B-cell-deficient mice also failed to alter the incidence of diabetes onset [91]. Notably, an early study using mice infected with 1 × 10^4^ PFU of EMCV-M suggested that the susceptibility to VID is determined by a single autosomal recessive gene inherited in a Mendelian fashion [92].

#### 3.1.1. Ifih1, Tlr3, Irf3, and Ifn-β

The partial loss of Ifih1 in mice with a B6 background and complete deficiency of Ifih1 in mice with a 129/SvJ background led to the development of transient hyperglycemia and overt diabetes after infection with 1 × 10^3^ PFU of EMCV-D, respectively [93]. The IFIH1-deficient mice showed reduced type 1 IFN production and increased viral titer in the pancreas after EMCV-D infection compared with wild-type mice. Mice with deficiency of Tlr3, a virus recognition molecule that senses dsRNAs in the endosomal compartment, showed an increased viral titer in the pancreas and the presence of EMCV antigen mainly in the islets, resulting in the development of diabetes [93]. Moreover, bone marrow (BM) chimeras containing Tlr3^−/−^ hematopoietic cells and wild type (WT) stroma developed diabetes after EMCV-D infection, whereas chimeras containing WT hematopoietic cells and Tlr3^−/−^ stroma were protected [93]. CD11c-positive DCs and possibly other Tlr3-positive myeloid cells may play a role in the Tlr3-mediated control of EMCV-D-induced diabetes [93]. In addition, interferon regulatory factor 3 (Irf3) and IFN-β-deficient mice developed diabetes after EMCV-D infection. Both BM chimeras containing Irf3- or Ifn-β-deficient hematopoietic cells and WT stroma developed diabetes. It was proposed that optimal viral sensing and type 1 IFN production from the host hematopoietic cells are required to prevent VID onset after β-cell-tropic virus infection [93].

#### 3.1.2. Tyk2

In our previous study, it was reported that mice with Tyk2 deficiency against a B6 background challenged with 1 × 10^3^ PFU of EMCV-D developed diabetes [94]. The irradiated Tyk2-deficient mice that were transferred spleen cells derived from either WT or Tyk2-deficient mice developed diabetes, whereas the irradiated WT mice that received spleen cells from either WT or Tyk2-deficient mice showed resistance. Moreover, the recovery of Tyk2 gene expression specific to β-cells in the Tyk2-deficient B6 mice was sufficient to prevent diabetes onset [94]. Although Tyk2-deficient mice with EMCV-D showed increased viral titer in the pancreas, increased IFN-α concentrations in serum and the pancreas were observed. In addition, high-dose IFN-α treatment after EMCV-D infection did not inhibit diabetes onset [94], suggesting that Tyk2-deficient mice were refractory to high-dose IFN. These observations suggested that Tyk2-mediated antiviral responses in the β-cells are critical for the prevention of VID.

These findings prompted us to examine the Tyk2 gene sequence in inbred mice. We found that EMCV-D induced diabetes in susceptible strains, SJL and SWR, harboring a mutated Tyk2 gene. The SNPs in the Tyk2 gene reduced the Tyk2 gene promoter activity and the antiviral gene expression in β-cells [94]. Notably, after stimulation with a high dose of IFN-β, mouse embryonic fibroblasts (MEFs) with mutated Tyk2 gene and Tyk2-deficient allele increased the survival after EMCV-D infection, whereas β-cells with mutated Tyk2 gene and Tyk2-deficient allele did not. Taking these findings together, we concluded that β-cell-specific Tyk2-mediated antiviral responses through type 1 IFN stimulation are important for preventing VID, and the Tyk2 gene is a gene variant represented in SJL and SWR mice that naturally confers susceptibility to EMCV-D-induced diabetes [94].

#### 3.1.3. Il-1β, Tnf-α, and iNOS

Mice treated with antibodies against IL-1β and TNF-α, or with inhibitor of inducible NO synthase (iNOS), showed a reduced incidence of VID upon infection with a low dose of EMCV-D [90]. iNOS gene-deficient DBA/2 mice showed reductions in IL-1β and TNF-α mRNA expression in both macrophages and β-cells, β-cell apoptosis, and the incidence of VID, indicating that NO plays an important role in the activation of macrophages and apoptosis of β-cells in the model with infection by a low dose of EMCV-D [88].

#### 3.1.4. Reg1

Regenerating (Reg) family members have been shown to promote proliferation and differentiation, and prevent apoptosis in diverse cell types in different contexts [95]. It was reported that the levels of serum REG1A were increased in diabetes patients [96]. Reg1 was found to be hyper-expressed after infection with 1 × 10^3^ PFU of EMCV-D in acinar-like cell clusters touching Langerhans islets with thin interstitial surrounding (ATLANTIS), as is the case with human EV-induced T1D [97,98]. Reg1-deficient mice exhibited a lower number of bromodeoxyuridine (BrdU)-positive β-cells than WT mice, suggesting that Reg1 has mitogenic or regenerative (or both) function in β-cells with virus-induced damage [97].

#### 3.1.5. Stat2

Our recent study suggested that impaired upregulation of the signal transducer and activator 2 (Stat2) gene only in β-cells is responsible for VID in DBA/2 mice with 1 × 10^3^ PFU of EMCV-D [99]. Transcriptomic analysis of β-cells purified from EMCV-D-infected mice showed that the innate immune responses were impaired in the β-cells derived from DBA/2 mice compared with that in the β-cells derived from B6 mice. Impaired upregulation of Stat2 gene expression was observed in β-cells derived from DBA/2 mice following EMCV-D infection or IFN-β administration. Other tissues such as spleen, liver, heart, and brain from DBA/2 mice showed upregulated Stat2 gene expression compared with those from B6 mice. Consistent with this finding, significantly high viral titers were observed only in the pancreas. Taken together, these data suggest that Stat2-mediated antiviral responses in β-cells are important for preventing EMCV-D-induced diabetes in DBA/2 mice [99], and that the analysis of β-cells in an in vivo model is important for elucidating the pathogenesis of VID.

### 3.2. KRV-Induced Autoimmune Diabetes Model

Kilham rat virus (KRV) is a nonenveloped DNA virus belonging to the parvovirus family. KRV was isolated from spontaneously diabetes-prone (DP)-BB rats by the plaque-purification method [30]. KRV induces autoimmune diabetes in diabetes-resistant (DR)-BB and LEW1.WR1 rats within 2–4 weeks post-infection [30,100]. After KRV infection, viral antigen was not observed in the islets, and β-cell cytolysis was not detected until lymphocyte infiltration occurred [30]. Contrary to these findings, a recent study reported that KRV was detected in the islets 5 days after infection [101]. The anti-inflammatory histone deacetylase inhibitor (HDACi) ITF-2357 reduced the T-cell accumulation in the spleen or pancreatic lymph nodes, resulting in a decreased incidence of diabetes [102]. Treatment with anti-T cell antibodies protected against the onset of diabetes in DR-BB and LEW1WR1 rats [100]; therefore, KRV-induced diabetes occurs in a T-cell-dependent manner.

#### 3.2.1. MHC (RT1)

Rat MHC is named RT1. A study using inbred and congenic rats suggested that class I A^u^ and class II B/D^u^ gene products, the MHC loci shared by DR-BB and LEW1.WR1, are required for KRV-induced diabetes [100]. It also suggested that the RT1^u^ haplotype alone is not sufficient for KRV-induced diabetes [100].

#### 3.2.2. Ifnar1

In IFN-α receptor 1 (Ifnar1)-deficient LEW.1WR1 rats, the incidence of KRV-induced diabetes was shown to be reduced [103]. These rats also showed a reduced incidence of poly(I:C)-induced autoimmune diabetes. The splenic cells from the Ifnar1-deficient rats with KRV showed reduced expression of C-X-C motif chemokine ligand (Cxcl) 10 and C-C motif chemokine ligand (Ccl) 5, whereas the expression of Il-1β and Ccl2 was increased [103]. These findings indicate that type 1 IFN-mediated signaling is associated with the development of autoimmune diabetes induced by KRV [103].

### 3.3. CVB-Induced Acute Autoimmune NOD Diabetes Model

CVB-infected NOD mice have been used to analyze VID. Because female NOD mice developed spontaneous autoimmune diabetes at a high rate [104,105], the role of virus infection in the CVB-infected NOD model is based on the autoimmunity to β-cells. In female NOD mice, the infiltration of immune cells starts at approximately 3 weeks of age [106]. T cells, B cells, DCs, macrophages, and neutrophils were detected in the inflamed islets [105,107,108]. Notably, in the CVB-infected NOD model, the time point of CVB infection was shown to influence the outcome. Specifically, young female NOD mice (approximately 4–6 weeks old, suggesting that the insulitis was not fully established) showed delayed and reduced incidence of T1D upon CVB infection, while older mice (approximately over 10 weeks old, suggesting the existence of a critical mass of autoreactive cells in the islets) showed the accelerated induction of T1D by CVB [20,109,110,111]. Delayed onset of diabetes in young NOD mice with CVB may be due to the transient upregulation of programmed cell death-1 ligand 1 (PD-L1) on lymphoid cells and an increased number of invigorated Tregs [112]. An accelerated onset of diabetes in older NOD mice with CVB may be due to the induction of bystander activation of autoreactive T cells [110]. Interestingly, in contrast to NOD mice with CVB, EMCV-D-infected young female NOD mice showed an accelerated onset of diabetes, whereas older NOD mice infected with EMCV-D showed a reduced incidence of diabetes [113]. These observations suggested that the timing of viral infection influences the outcome may depend on the virus strain. It was also reported that the diabetogenic phenotype of CVB correlates with its replication efficiency in β-cells [114].

#### 3.3.1. Tlr3

Eight-week-old toll-like receptor 3 (Tlr3)-deficient female NOD mice showed reductions in the insulitis score and the incidence of T1D upon CVB4 infection [115]. These findings demonstrate that Tlr3-mediated CVB recognition is critical for accelerating the onset of diabetes in genetically prone NOD mice [115].

#### 3.3.2. Ifih1

Ten- to twelve-week-old female NOD mice heterozygous for the Ifih1 gene showed complete protection against the onset of diabetes following CVB4 infection, in contrast to their wild-type littermates in which the incidence of diabetes reached 50% [116]. In contrast, the deficiency or partial loss of Ifih1 expression reduced the incidence of spontaneous autoimmune diabetes in female NOD mice. The partial loss of Ifih1 gene expression altered IFN-β production levels and regulatory T-cell (Treg) responses after CVB4 infection, suggesting that altering Ifih1 expression regulates the autoreactive component of the host response to infection that protects against the onset of VID [116].

#### 3.3.3. Socs-1

Suppressor of cytokine signaling 1 (Socs-1) plays a role in negative regulation of the Jak-Stat pathway, IFN-γ secretion, anti-tumor immunity, and infection immunity [117,118,119]. Transgenic (Tg) female NOD mice (8–9 weeks old) that expressed Socs-1 specific to β-cells developed acute diabetes by CVB4 infection [119]. Socs1-overexpressing β-cells could not inhibit CVB4 replication in response to IFN-α. Adaptive immunity is not necessary for the swift induction of diabetes, but depletion of NK cells prevents diabetes. These findings showed that an intact β-cell response to type 1 IFN stimulation is required to prevent NK cells from attacking β-cells and the onset of diabetes after CVB4 infection [119]. It is suggested that even in a host background prone to autoimmunity, the antiviral defense in β-cells plays a role in preventing VID onset. The Socs-1 Tg NOD mice have been used as a model animal to verify the effects of vaccination [120,121].

### 3.4. Transgenic Animal Models

Transgenic mice expressing lymphocytic choriomeningitis viral (LCMV) glycoprotein (GP) or nucleoprotein (NP) under the control of rat insulin promoter (RIP) (RIP-LCMV) developed autoimmune diabetes following LCMV challenge [29,122]. There are two models of these mice: a rapid-onset model (RIP-GP line), in which the LCMV transgene is expressed only in β-cells and diabetes develops within 2 weeks post-infection; and a slow-onset model (RIP-NP line), in which the LCMV transgene is expressed in the pancreas and thymus and diabetes develops within 2 months post-infection in H-2^d^ mice or within 3–6 months post-infection in H-2^b^ mice [86,122]. For details of the other transgenic mouse models expressing influenza viral protein hemagglutinin (HA) under the control of RIP [123] or expressing human Epstein–Barr virus (EBV) receptor CR2 (CD21) under the control of RIP [124], see a previous review [86].

#### 3.4.1. Ifn-α

In the rapid-onset RIP-LCMV model (RIP-GP), although viral titer was increased, treatment of anti-Ifn-α or anti-Ifnar antibodies completely prevented the incidence of diabetes after LCMV infection, but treatment with anti-Ifn-β antibody did not [125]. The sphingosine-1-phosphatase receptor 1 (S1PR1) agonist, which promotes the turnover of Ifnar at the cell surface and inhibits type 1 IFN signaling, also reduced the incidence of diabetes onset. Blockade of Ifn-α signaling reduced the migration of autoreactive T cells into the islets [125]. These findings suggested that Ifn-α signaling is critical to the development of autoimmune diabetes. Recently, it has been suggested that viruses act by causing a type 1 interferonopathy within the infected pancreas and the microenvironment of the islets, resulting in T1D [126]. Type 1 interferonopathy has been proposed to overlap with T1D [126], and therefore the host genetic factor(s) conferring a tendency to produce high levels of type 1 IFN by viral infection may be associated with the pathogenesis of VID.

#### 3.4.2. Cxcl10

The C-C motif chemokine ligand (Cxcl) 10-deficient islet isografts from RIP-LCMV mice prevented autoimmune attack despite engrafting on LCMV-infected hosts [127]. The administration of Cxcl10 antibody also prevented the autoimmune destruction of engrafted islets. RIP-NP mice crossed with RIP-Cxcl10 mice developed diabetes within 4 weeks after LCMV infection, and autoaggressive antigen-specific CD8 T cells were observed in the islets of the diabetic mice [128]. Consistent with these findings, islet cells of fulminant T1D patients strongly expressed CXCL10 [7]. A recent study showed that the induction of Cxcl10 was mediated by calcineurin-dependent nuclear factor of activated T cells (NFAT) signaling in β-cells in response to oxidative or inflammatory stress [129]. These observations suggested that Cxcl10 plays an important role in the recruitment of autoreactive lymphocytes into the islets in response to diabetogenic virus infection [7,127,128].

### 3.5. Combined STZ and Virus Infection Model

STZ induces β-cell injury in a dose-dependent manner, resulting in β-cell destruction and diabetes upon reaching a certain diabetogenic dose level. It was reported that mice treated with a sub-diabetogenic dose of STZ developed diabetes following virus infection. EMCV-D could induce diabetes in STZ-treated resistant strains of mice such as C3H, C57BL/6, CBA, and AKR [130]. CVB3, CVB5, and CVB4 also induced diabetes in mice treated with a sub-diabetogenic dose of STZ [130,131]. These observations suggest that β-cell injury or exhaustion caused by environmental factors such as STZ may increase the risk of VID onset. In another paper, viral diabetogenicity was tested in a model combining STZ and virus infection [132]. Further studies using models combining STZ and virus infection should open up perspectives to better understand the interactions between the virus and host [131].

## 4. Conclusions

**Figure 1:**
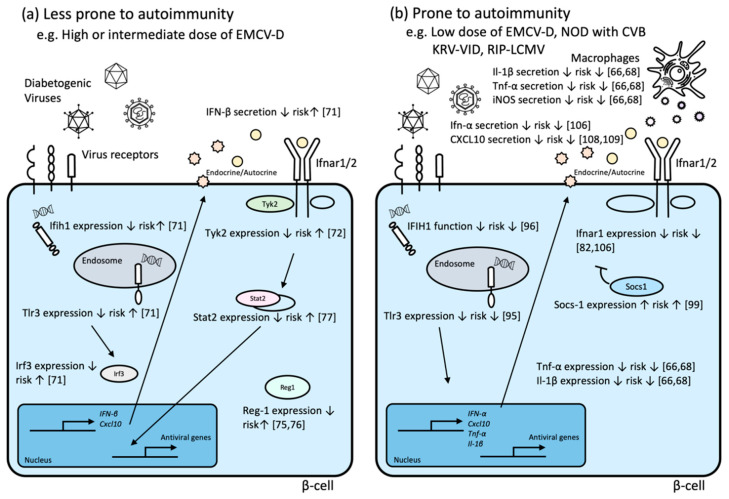
Virus-induced diabetes-associated genes in animal models reviewed in this article. (**a**) These candidate genes were reported from studies of hosts that are less prone to autoimmunity. (**b**) These candidate genes were reported from studies of hosts that are prone to autoimmunity. The numbers in square brackets are reference article numbers.

The results obtained from experiments in humans and animal models clearly indicated that host genetic factors have an impact on the development of VID (Table 2, Figure 1).

The effects of the individual SNPs in T1D have been reported in human studies, while the combined effects of individual SNPs in humans remain unclear. On the other hand, in animal models, Gorman J.A. et al. showed that mice with the two human autoimmune risk variants, IFIH1 (rs1990760) and PTPN22 (rs2476601), increased the incidence of autoimmune diabetes by low-dose STZ challenge more than those with non-risk allele or those with one risk allele, demonstrating that an additive effect of risk alleles could promote the development of T1D [51]. These data provided the importance of mouse modeling to assess the effect of candidate GWAS variants and the combinational effects of SNPs, as disease associated genetic variants probably do not operate in isolation [51]. As reviewed in this paper, the effect of some SNPs depends on tested populations. Additionally, the role of genes in VID animal models depends on host background (Figure 1). In addition, the combination effect of SNPs should be considered as shown in animal models [51]. These data imply that the magnitude of interaction between T1D and environmental factors, at least in part, depends on studied populations. Further studies in different human populations, VID models with different background of animal strains, and animal models with multiple human T1D risk alleles may be needed to elucidate further interactions between virus infection and genetics of T1D.

Although Enteroviruses, especially the Coxsackie B virus, are considered to be some of the major diabetogenic agents in humans, other viruses are also potential causal agents (Table 1). The comparison of the expression levels of HCAR and the other viral receptors on the surface of human islet cells might contribute to the further understanding of the viral diabetogenicity and the specific association of virus groups with T1D in humans.

**Figure 2:**
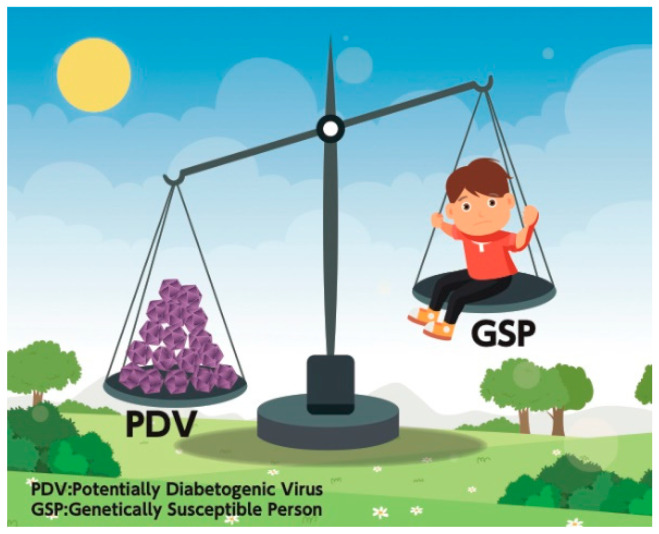
Balance between diabetogenicity of the virus and genetical susceptibility of the host. A potentially diabetogenic virus may cause diabetes in a genetically susceptible person. (The figure is derived from the front cover of the Journal of Medical Virology, August issue 2019 [134], following minor modification with permission from the publisher.)

Arguably, host background, in terms of whether the host is prone to autoimmunity, should also be considered, because the role of genes depends on host background. We propose the importance of dissecting the in vivo VID models because virus infection elicits cell-mediated immunity, humoral immunity, cell death, and rapid changes of gene expression in host cells including β-cells [99]. Notably, according to some studies, the β-cell-specific gene regulation and response to infectious agents may have a major influence on the development of VID. On the basis of the VID animal models described above, animals harboring VID-associated SNPs may deepen our understanding of the host genetic factors and the pathogenesis of VID. Moreover, these animal models can help in identifying diabetogenic or latently diabetogenic (or both) viruses in humans (Figure 2) [133], leading to the development of novel vaccines to prevent VID [134].

## Figures and Tables

**Table 1:** List of potential diabetogenic viruses in humans and animals.

**Humans**		
	Virus	Ref.
RNA virus	Enterovirus	[7,8]
	Rotavirus	[9]
	Retrovirus	[10,11,12]
	Rubella virus	[13,14]
	Mumps virus	[6]
DNA virus	Cytomegalovirus	[15,16]
	Epstein-Barr virus	[17]
	Parvovirus	[18]
	Herpesvirus	[19]
**Animals**		
	Virus	Ref.
RNA virus	Enterovirus	[20]
	Encephalomyocarditis virus	[21]
	Mengovirus	[22]
	Rotavirus	[23]
	Retrovirus	[24]
	Reovirus	[25]
	Rubella virus	[26]
	Influenza A virus	[27]
	Bovine viral diarrhea-mucosal disease virus	[28]
	Lymphocytic choriomeningitis virus	[29]
DNA virus	Kilham rat virus (Rat Parvovirus)	[30]
	Cytomegalovirus	[31]

**Table 2:** List of potential Single Nucleotide Polymorphisms (SNPs) associated with human Virus-Induced Diabetes (VID).

Candidate Genes	SNPs	Nucleoside Change (Amino Acid Change)	The Effect of the SNPs (OR, Odds Ratio; HR, Hazard Ratio)	Potential Association with VID
IFIH1	rs1990760	NC_000002.12:g.162267541C>T (NP_071451.2:p.Ala946Thr)	T1D, HR 1.21 [37]; EV prevalence in stools, OR 1.22 [38]; The association with islet autoimmunity, OR 1.2 [38]	Linked with the high expression of type III IFNs but not with IFN-β in pancreatic islets from human donors following CVB3 infection [39]; Associated with the production of β-cell autoantibodies (IAA, GADA, IA-2A) but not the development of T1D in Finnish population (Finnish DIPP study) [37].
	rs35667974	NC_000002.12:g.162268127T>C (NP_071451.2:p.Ile923Val)	T1D OR 0.59 [40]; EV prevalence in stools, OR 0.86 [38]	Association was not observed with the frequency of EV RNA in stools from Norwegian newborns [38].
	rs35337543	NC_000002.12:g.162279995C>G; NC_000002.12:g.162279995C>T	EV prevalence in stools, OR 0.78 [38]	Association was not observed with the frequency of EV RNA in stools from Norwegian newborns [38].
	rs35744605	NC_000002.12:g.162277580C>A, C>G, C>T; (NP_071451.2:p.Glu627Ter, Gln, Lys)	EV prevalence in stools, OR 0.90 [38]	Association was not observed with the frequency of EV RNA in stools from Norwegian newborns [38].
	rs35732034	NC_000002.12:g.162268086C>T	EV prevalence in stools, OR 2.47 [38]	Marginally increased prevalence of EV RNA was observed in stools from Norwegian newborns [38].
TYK2	rs2304256	NC_000019.10:g.10364976C>A (NP_003322.3:p.Val362Phe)	-	Associated with EVB presence in stools, but not with the presence of islet autoantibodies in the United States and European population [41]; Association was not observed with the presence of flu-like syndrome at T1D onset in Japanese population [42].
	rs2304258, rs17000728, rs17000730, rs2304259, rs891696485, rs953883300	NC_000019.10:g.10380510C>T; NC_000019.10:g.10380511C>T; NC_000019.10:g.10380572T>C; NC_000019.10:g.10380676T>G; NC_000019.10:g.10381501A>C, A>T; NC_000019.10:g.10381502C>T	T1D, OR 2.4 [42]; T1D with flu-like syndrome, OR 3.6 [42]; T1D without GADA, OR 3.3 [42]; T2D, OR 2.1 [42]; T1D, OR 1.89; T1D, HR 1.21 [37]	These SNPs, named TYK2 promoter variant (ClinVar, 440728), showed the risk of T1D, especially high risk in T1D associated with flu-like syndrome at the onset and those with GADA negative in Japanese population [42,43].
CXADR	rs6517774	NC_000021.9:g.17449955A>G	The association with islet autoimmunity, OR 1.47 [41]	The lower detection of EVB in stools in the United States and European population [41].
PTPN22	rs2476601	NC_000001.11:g.113834946A>G (NP_057051.3:p.Arg620Trp)	T1D, OR 1.89 [40]; T1D, OR 1.96 [44]; T1D, HR 1.97 [37]; The association with islet autoimmunity, OR 1.99 [41]	The prolonged shedding or consecutive positive for EVB, HAdV-C, HAdV-F in stools in the United States and European population [41].

The SNP data were obtained from the Single Nucleotide Polymorphism Database (dbSNP). These data were based on Genome Reference Consortium human build 38 patch release 12 (GRCh38.p12).
